# Recording of Severe Mental Illness in United Kingdom Primary Care, 2000–2010

**DOI:** 10.1371/journal.pone.0082365

**Published:** 2013-12-12

**Authors:** Sarah Hardoon, Joseph F Hayes, Ruth Blackburn, Irene Petersen, Kate Walters, Irwin Nazareth, David P. J. Osborn

**Affiliations:** 1 Research Department of Primary Care and Population Health, University College London, London, United Kingdom; 2 Mental Health Sciences Unit, University College London, London, United Kingdom; University of Hertfordshire, United Kingdom

## Abstract

**Background:**

There is increasing emphasis on primary care services for individuals with severe mental illnesses (SMI), including schizophrenia, bipolar disorder, and other non-organic psychotic disorders. However we lack information on how many people receive these different diagnoses in primary care. Primary care databases offer an opportunity to explore the recording of new SMI diagnoses in representative general practices.

**Methods:**

We used data from The UK Health Improvement Network (THIN) primary care database including longitudinal patient records for individuals aged over 16 years from 437 general practices. We determined the annual GP recorded rate of first diagnosis of SMI by age, gender, social deprivation and urbanicity between 2000 and 2010.

**Results:**

We identified 10,520 individuals with a first record of schizophrenia, bipolar disorder or other non-organic psychosis among 4,164,794 patients. This corresponded to a rate of first diagnosis of 46.4 per 100,000 person years at risk (PYAR) (95% CI 45.4 to 47.4) in the 16–65 age group. The rate of first record of schizophrenia was 9.2 per 100,000 PYAR (95% CI 8.7 to 9.6) in this age group, bipolar disorder was 15.0 per 100,000 PYAR (95% CI 14.4 to 15.5) and other non-organic psychotic disorder was 22.3 per 100,000 PYAR (95% CI 21.6 to 23.0).

**Conclusions:**

The rates of GP recorded SMI in primary care records were broadly comparable to incidence rates from previous epidemiological studies of SMI and show similar patterns by socio-demographic characteristics. However there were some differences by specific diagnoses. GPs may be recording rates that are higher than those used to commission services.

## Introduction

Individuals with Severe Mental Illness (SMI); defined as schizophrenia, bipolar disorder and other non-organic psychotic conditions, in keeping with the Quality Outcomes Framework [1], constitute around 2% of the population (lifetime prevalence) [2]. Recent epidemiological studies have confirmed that SMI incidence is related to sex, migrant status, urbanicity, season of birth, and economic status [3]–[5], but it is now clear that there is significant heterogeneity across populations [6]–[8]. A recent meta-analysis of English studies from 1950–2009 showed a pooled incidence of 31.7 per 100,000 person years at risk (PYAR) for all psychotic illness[8] with a range from 21 to 50 per 100,000 PYAR [9], [10] Specifically, schizophrenia incidence is around 15 per 100,000 PYAR [11] and affective psychosis 12 per 100,000 PYAR [8].

Accurate incidence data are vital for planning service provision in both primary and secondary care [12], for understanding any underlying changes in the SMI population over time, and to contextualise and validate SMI research which utilises primary care clinical data. However we lack contemporary information on SMI incidence rates in the UK, either in the community or in primary care settings. There is one existing study using primary care data to examine changes in new recording of psychotic disorder [13]. This study suggested rates remained stable over a ten-year period (1996–2005) and its inclusion criteria (which include chronic disorders and symptoms such as “paranoia”) are likely to have produced an overestimate of SMI.

In the United Kingdom, the care of people with SMI is included in the GP contract [1]. Since 2004 this has been included in *Quality and Outcomes Framework* (QOF), whereby practices receive remuneration for keeping a register of people who have a diagnosis of SMI and for offering them an annual review. The implementation of QOF should mean that SMI is recognised and recorded more frequently. Care may be provided by the GP alone or in conjunction with secondary services, either Early Intervention Services (traditionally for under 35 year-olds within three years of a first episode of psychosis) or General Adult Services (under 65 years old) [14].

Our main objective was to estimate number of individuals with a new record of schizophrenia, bipolar disorder and other non-organic psychotic conditions in primary care annually between 2000 and 2010, using data from The Health Improvement Network (THIN) database, a UK primary care database. We also aimed to examine the recording of these diagnoses by socio-demographic factors such as age, gender, social deprivation and urbanicity.

## Materials and Methods

### Data Source

The Health Improvement Network (THIN) database is one of the largest UK sources of continuous primary care data, containing information on illness recording and prescribing behaviour. At the time of data collection from THIN (http://csdmruk.cegedim.com) 437 participating general practices, contributing more than 10 million patients, were included. The database is broadly representative of the UK population [15]. In the UK most people with SMI are registered with primary care [16] and the validity of general practice computer diagnoses of SMI has been established previously [17]. THIN data have also been shown to be roughly representative of UK general practice in terms of consultations and prescribing statistics [18], [19]. THIN contains records of each patient's medical conditions and symptoms, recorded during routine consultations and all prescriptions issued by GPs. Symptoms and diagnoses are classified using the Read code system, a hierarchical recording system used to record clinical summary information [20]. This creates a computerised medical history for each patient from the time they register with a general practice. In addition, the database holds information on basic demographics, urbanicity and social deprivation. Based on their residential postcode, patients are classified as residing in urban areas (population >10,000); or in towns and fringes; or in villages, hamlets and isolated areas. Social deprivation is measured using the Townsend score for the postcode sector area of residence, linked to population census data from 2001 [21]. It is a combined measure of owner-occupation, car ownership, overcrowding and unemployment [22]. The scores are defined for small areas of around 150 households, and grouped into quintiles.

### Ethics Statement

The scheme for THIN to obtain and provide anonymous patient data to researchers was approved by the National Health Service South-East Multicentre Research Ethics Committee (MREC) in 2002 and scientific approval for this study was obtained from CMD Medical Research's Scientific Review Committee in March 2012.

### Study Population

We included data from the date at which practices had met quality assurance criteria, namely continuously acceptable computer usage (ACU) (i.e. one medical record, one additional health data record per patient per year, and at least two prescriptions, on average per patient per year [23]) and the criteria for acceptable mortality reporting (AMR) which indicate a point at which the observed death rate for a practice corresponds to that expected based on predicted numbers of deaths derived from National statistics given the practice's demographics [24], [25].

We included all individuals aged 16 to 95 years, permanently registered for at least one year during the period from 1 January 2000 to 31 December 2010. We examined two subgroups according to age criteria: those traditionally eligible for entry to Early Intervention Services in the UK (16 to 35 years old)[26], [27] those eligible for entry to General Adult psychiatric services (16 to 65 years old).

For this study we were interested in individuals who had a first recording suggestive of a new diagnosis of SMI in their primary care records. Therefore we excluded patients who had a record of SMI prior to start of follow-up (see details of follow-up in statistical analysis) or whose first SMI record during follow-up was indicative of pre-existing SMI or repeated episodes (such as chronic paranoid schizophrenia, or manic relapse). We also excluded diagnoses made within the first year of registration, as people who received a code within the first year were more likely to be prevalent rather than incident cases [28].

### Measurement of main outcome

Cases of SMI included those who had new records of a Read code for SMI (schizophrenia, bipolar disorder, other non-organic psychotic illness). A list of all SMI diagnoses was constructed using established methods [29] and cross-checked with lists of codes given in national QOF guidance.

SMI Patients were classified according to the type of diagnosis (schizophrenia, bipolar disorder, other non-organic psychosis). If patients first received a code for “other psychosis”, but had subsequent codes to indicate schizophrenia or bipolar disorder, they were reclassified as schizophrenia or bipolar accordingly. However, the date of the first diagnosis was retained as the date of the first record. Similarly, if patients first received a code to indicate inclusion on an SMI register, they were reclassified as schizophrenia, bipolar or other psychosis if they received these diagnoses subsequently. If patients received both bipolar and schizophrenia diagnoses, they were coded as their latest diagnosis (as this was considered likely to be most accurate, having considered the whole longitudinal medical history). Patients with no diagnostic codes at any time but with codes to indicate inclusion on an SMI register were excluded from the analysis. Patients receiving a diagnosis code of dementia within a year of their SMI code were excluded.

The number of individuals with a newly recorded diagnosis was determined by age (10 year age groups and service-line groups), sex, urbanicity and quintiles of Townsend score. Individual level ethnicity was not well recorded historically in THIN, therefore it was not possible to describe recording by this covariate.

### Statistical Analysis

The recording of coded SMI was estimated per 100,000 person years at risk (PYAR) as the total number of new SMI cases recorded between 2000 and 2010, divided by the total number of person years of follow-up. Person-time for the denominator was estimated as the latest of: [16^th^ birthday, one year's registration, ACU/AMR date, start date of period], to the earliest of: [date of first incident diagnosis, date of death, date patient leaves practice, date of last data collection from the practice, end date of period].Recorded rates of all SMI and of different forms of SMI (schizophrenia, bipolar disorder, other psychosis) were estimated, according to age, gender, Townsend score and urbanicity.

Annual rates were graphed to examine the time trends. Multivariable Poisson regression models with (log) person-time as an offset, were used to examine recording of all SMI by gender, age (in 10 year age-bands) deprivation (quintiles of Townsend scores) and urbanicity (as three categories: urban, town/fringe and village/hamlet/isolated). Multilevel random intercept models were used to account for clustering of patients in practices. All analyses were carried out using STATA 12.

## Results

In total, 10,520 individuals (amongst 4,164,794 patients) had an electronic record indicating that they had a new diagnosis of severe mental illness between 2000 and 2010. This was equivalent to 44.9 per 100,000 PYAR (95% Confidence Interval (CI) 44.0 to 45.7). There were substantial differences by age and sex within different diagnoses. Of the diagnoses made, 18% were classified as schizophrenia, 30% bipolar disorder and 52% other non-organic psychotic disorder. For the 16–65 age group (eligible for General Adult Services) the rate of recording was 46.4 per 100,000 PYAR (95% CI 45.4 to 47.4) and for the 16–35 age group (eligible for Early Intervention Services) the rate of newly recorded diagnoses cases was 58.1 per 100,000 PYAR (95% CI 56.3 to 60.0).

### Schizophrenia

**Figure 1:**
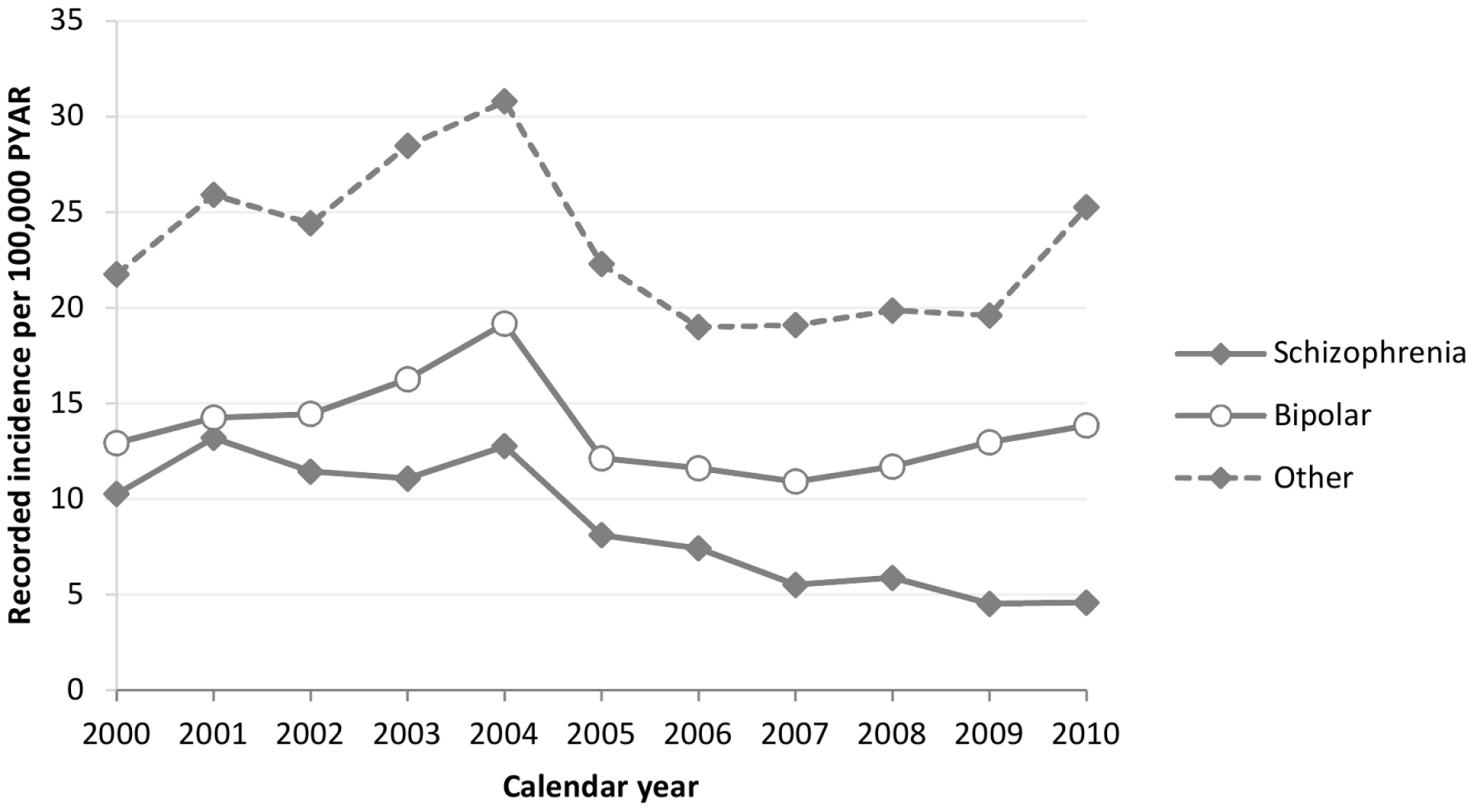
Time trends in GP recorded diagnosis of schizophrenia, bipolar disorder and other psychosis.

Schizophrenia was the least commonly recorded diagnosis of severe mental illnesses in primary care records. Up to 2004, there were between 10 and 14 new entries per 100,000 PYAR. However, by 2007 there were only around 5 new entries per 100,000 PYAR (Figure 1). In the 16–65 age group the rate was 9.2 per 100,000 PYAR (95% CI 8.7 to 9.6) and in the 16–35 age group it was 14.3 Per 100,000 PYAR (95% CI 13.4 to 15.3). Schizophrenia was more commonly recorded in men than women (adjusted IRR 0.6, 95% CI 0.54 to 0.66) (Table 1). In men the diagnosis was most commonly recorded in the 16–24 age group and recording reduced with increasing age, whereas in women there was no difference across age groups after adjustment for other factors (Table 2). Recording of schizophrenia increased with increasing social deprivation such that individuals in the most deprived quintile of Townsend score were nearly 5 times more likely to receive a diagnosis of schizophrenia, than those in the least deprived quintile (Table 1). After accounting for age, sex and social deprivation there was no difference in recording of schizophrenia in urban versus rural areas (Table 1).

**Table 1:** Recording rate of individual diagnoses by socio-demographic factors.

	Schizophrenia			Bipolar disorder			Other psychosis		
	Rate per 100,000 PYAR (95% CI)	Adjusted* IRR (95% CI)	p	Rate per 100,000 PYAR (95% CI)	Adjusted* IRR (95% CI)	p	Rate per 100,000 PYAR (95% CI)	Adjusted* IRR (95% CI)	p
**Overall**	8.2 (7.9 to 8.6)			13.6 (13.1 to 14.0)			23.1 (22.5 to 23.7)		
**Gender**			<0.001			<0.001			0.3
Men	10.4 (9.8 to 11.0)	1		10.9 (10.3 to 11.5)	1		22.5 (21.6 to 23.4)	1	
Women	6.2 (5.7 to 6.6)	0.60 (0.54 to 0.66)		16.1 (15.4 to 16.9)	1.50 (1.38 to 1.62)		23.7 (22.8 to 24.6)	1.03 (0.97 to 1.09)	
**Townsend quintile**			<0.001			<0.001			<0.001
1 (Least deprived)	3.7 (3.2 to 4.2)	1		10.0 (9.3 to 10.8)	1		13.7 (12.8 to 14.6)	1	
2	4.7 (4.2 to 5.4)	1.30 (1.08 to 1.57)		12.2 (11.3 to 13.2)	1.20 (1.07 to 1.35)		17.1 (16.0 to 18.2)	1.23 (1.11 to 1.36)	
3	7.3 (6.6 to 8.1)	1.81 (1.51 to 2.16)		14.0 (13.0 to 15.1)	1.39 (1.24 to 1.56)		22.3 (21.0 to 23.7)	1.53 (1.39 to 1.69)	
4	11.8 (10.8 to 12.9)	2.85 (2.40 to 3.39)		16.7 (15.5 to 18.1)	1.63 (1.45 to 1.84)		30.1 (28.5 to 31.9)	2.01 (1.83 to 2.22)	
5 (Most deprived)	21.8 (20.1 to 23.6)	4.75 (3.98 to 5.67)		18.8 (17.2 to 20.5)	1.84 (1.61 to 2.12)		46.8 (44.2 to 49.4)	2.93 (2.64 to 3.25)	
**Urbanicity**			>0.9			0.7			0.2
Urban	8.6 (8.1 to 9.1)	1		13.6 (13.0 to 14.2)	1		23.9 (23.1 to 24.7)	1	
Town and fringe	8.0 (7.1 to 8.9)	1.00 (0.85 to 1.18)		13.5 (12.4 to 14.7)	1.02 (0.91 to 1.15)		20.7 (19.3 to 22.2)	0.93 (0.83 to 1.03)	
Village/hamlet/isolated	4.5 (3.6 to 5.6)	0.96 (0.75 to 1.24)		10.5 (9.0 to 12.1)	0.95 (0.80 to 1.12)		15.9 (14.1 to 17.9)	0.89 (0.77 to 1.02)	

from multilevel Poisson regression, with patients nested in practices, adjusting for the other variables considered.

**Table 2:** Recording of rate of individual diagnosis by age and gender.

	Schizophrenia				
	Rate per 100,000 PYAR (95% CI)		Adjusted* IRR (95% CI)		p^†^
Age, years	Men	Women	Men	Women	<0.001
16–24	24.7 (22.3 to 27.4)	7.3 (6.0 to 8.9)	1	1	
25–34	17.1 (15.3 to 19.2)	7.9 (6.7 to 9.4)	0.67 (0.57 to 0.78)	1.17 (0.88 to 1.56)	
35–44	10.0 (8.8 to 11.4)	6.6 (5.6 to 7.8)	0.42 (0.35 to 0.50)	1.08 (0.81 to 1.44)	
45–54	7.1 (6.0 to 8.3)	5.4 (4.5 to 6.6)	0.30 (0.24 to 0.37)	0.90 (0.67 to 1.23)	
55–64	4.5 (3.6 to 5.5)	4.5 (3.6 to 5.6)	0.21 (0.16 to 0.27)	0.77 (0.55 to 1.07)	
65–74	3.6 (2.7 to 4.8)	5.0 (4.0 to 6.4)	0.15 (0.11 to 0.22)	0.82 (0.58 to 1.15)	
75–84	2.9 (1.9 to 4.4)	6.3 (4.9 to 8.0)	0.13 (0.08 to 0.21)	0.96 (0.68 to 1.36)	
85–94	4.4 (2.2 to 8.8)	7.2 (5.0 to 10.4)	0.19 (0.09 to 0.39)	0.91 (0.56 to 1.47)	

	Bipolar disorder				
Age, years	Men	Women	Men	Women	<0.001
16–24	10.1 (8.6 to 11.9)	13.4 (11.5 to 15.5)	1	1	
25–34	12.0 (10.5 to 13.7)	22.5 (20.4 to 24.9)	1.23 (0.98 to 1.54)	1.59 (1.32 to 1.93)	
35–44	13.6 (12.2 to 15.2)	21.6 (19.8 to 23.7)	1.39 (1.13 to 1.73)	1.54 (1.28 to 1.86)	
45–54	12.9 (11.4 to 14.6)	19.0 (17.2 to 21.0)	1.34 (1.07 to 1.66)	1.40 (1.16 to 1.70)	
55–64	9.2 (7.9 to 10.7)	12.7 (11.2 to 14.5)	1.00 (0.78 to 1.27)	0.99 (0.80 to 1.22)	
65–74	8.3 (6.8 to 10.0)	10.2 (8.6 to 12.0)	0.87 (0.67 to 1.15)	0.73 (0.57 to 0.93)	
75–84	6.2 (4.6 to 8.3)	9.3 (7.6 to 11.4)	0.65 (0.46 to 0.93)	0.69 (0.53 to 0.90)	
85–94	6.0 (3.3 to 10.9)	5.2 (3.4 to 8.0)	0.57 (0.29 to 1.13)	0.41 (0.26 to 0.65)	

	Other Psychosis				
Age, years	Men	Women	Men	Women	<0.001
16–24	41.2 (38.0 to 44.6)	21.7 (19.4 to 24.4)	1	1	
25–34	30.5 (28.0 to 33.1)	23.0 (20.9 to 25.4)	0.73 (0.64 to 0.83)	1.01 (0.86 to 1.20)	
35–44	22.4 (20.5 to 24.4)	22.1 (20.3 to 24.2)	0.55 (0.48 to 0.63)	1.05 (0.90 to 1.24)	
45–54	16.3 (14.7 to 18.2)	21.0 (19.1 to 23.1)	0.42 (0.36 to 0.48)	1.04 (0.88 to 1.22)	
55–64	12.8 (11.3 to 14.6)	16.2 (14.5 to 18.2)	0.34 (0.29 to 0.41)	0.82 (0.69 to 0.98)	
65–74	12.8 (11.0 to 15.0)	21.4 (19.1 to 24.0)	0.34 (0.28 to 0.41)	1.06 (0.89 to 1.26)	
75–84	21.4 (18.3 to 25.0)	36.1 (32.6 to 40.0)	0.55 (0.45 to 0.67)	1.73 (1.47 to 2.05)	
85–94	31.9 (24.6 to 41.2)	65.3 (57.8 to 73.7)	0.77 (0.57 to 1.03)	3.10 (2.59 to 3.71)	

from multilevel Poisson regression, with patients nested in practices, adjusting for the other variables considered.

†for age-gender interaction.

### Bipolar disorder

Recording a new diagnosis of bipolar disorder ranged between 11 and 19 per 100,000 PYAR between 2000 and 2010, with a peak around 2004. However, by 2010 nearly 3 times as many people had a new record of bipolar as of schizophrenia (Figure 1) In the 16–65 and 16–35 subgroups bipolar disorder was recorded at a rate of 15.0 per 100,000 PYAR (95% CI 14.4 to 15.5) and 14.8 per 100,000 PYAR (95% CI 13.9 to 15.8) respectively over the period of study. In contrast to schizophrenia recording of bipolar disorder was more commonly recorded in women than men (Table 1). For men the first diagnosis was commonly recorded between the ages of 35–44 years, whereas women were diagnosed earlier (most commonly between 25–34 years) (Table 2). Like schizophrenia, recorded bipolar disorder increased with increasing social deprivation and the most deprived quintile was almost twice as common as the least deprived (Table 1). After accounting for age, sex and social deprivation there was no difference in recording of bipolar disorder in urban versus rural areas (Table 1).

### Other psychosis

Compared to schizophrenia and bipolar disorders a larger group had a record of non-organic psychotic illnesses (Figure 1). An increasing number of individuals received such diagnosis between 2000 and 2004. Thereafter, the recording fell to around 20 per 100,000 person years with an increase in 2010. It was recorded at a rate of 22.3 per 100,000 PYAR (95% CI 21.6 to 23.0) in the 16–65 age group, and 29.1 per 100,000 PYAR (95% CI 27.8 to 30.4) in the 16–35 age group. Similar to schizophrenia, recording was highest for men in the 16–24 age group, but for women the recording increased over 75 years old (Table 2). Again there was an increase in recording with increasing deprivation; with nearly three times as many individuals in the most deprived group, and no statistically significant relationship with urbanicity (Table 1).

### Diagnosis stability

For the majority of individuals with a record of SMI (90.3%) there was no discrepancy in diagnosis codes assigned over the 10 year study period (Table 3). However for those who had an initial record of non-organic psychosis 8.0% were subsequently coded as schizophrenia and 3.4% for bipolar disorder. A switch in code from bipolar to schizophrenia or vice versa occurred in 1.5% of the individuals (Table 3).

**Table 3:** Changes in diagnoses code.

		Diagnosis ultimately assigned* N (%)				
		Schizophrenia	Bipolar	Other	SMI register	Total
**First record of SMI**	Schizophrenia	1,257 (98.5)	19 (1.5)	0 (0)	0 (0)	**1,276**
	Bipolar	42 (1.5)	2,703 (98.5)	0 (0)	0 (0)	**2,745**
	Other	468 (8.0)	198 (3.4)	5,144 (88.5)	0 (0)	**5,809**
	SMI register	163 (3.4)	257 (5.3)	270 (5.6)	4,151 (85.8)	**4,841**
	**Total**	**1,930**	**3,177**	**5,413**	**4,151**	**14,671**

* Among patients whose first SMI record is for other psychosis, those who subsequently received a diagnosis of bipolar disorder or schizophrenia are re-classified as such. Among patients whose first SMI record indicates inclusion on an SMI register, those who subsequently received a diagnosis of schizophrenia, bipolar disorder or other psychosis are re-classified as such. Patients whose first record is for schizophrenia are re-classified as bipolar disorder if they subsequently received a bipolar disorder diagnosis and vice-versa (since the more recent record may be seen as the most accurate diagnosis).

## Discussion

We present data on over 10,000 newly recorded SMI diagnoses (schizophrenia, bipolar disorder and other non-organic psychosis) in routine primary care settings across the UK between 2000 and 2010. Over this time, recorded rate of all SMI among those aged 16 to 94 years was 44.9 per 100,000 PYAR (95% CI 44.0 to 45.7).

This study is the first to provide data on rate of recorded schizophrenia, bipolar disorder and other non-organic psychotic disorders in a large cohort of people seen in primary care over time. A recent meta-analysis [8] examining incidence in individuals under 65 years old, highlights the heterogeneity of incidence rates in psychotic disorders in England. Our recorded SMI rate for 16–65 year olds (46.4 per 100,000 PYAR, 95% CI 45.4 to 47.4) falls just above the confidence intervals of the incidence rate of all forms of psychotic illness in this meta-analysis (24.6 to 40.9 per 100,000 PYAR). Table 4 highlights how our findings fit with previous studies. Many previous studies examined incidence rate of first episode of psychosis in secondary care (with varying definitions of age of onset such as “first presentation”, “first contact” and “hospitalisation”, or a retrospective onset date), and often in a particular subgroup (for example those engaged in, or referred to, Early Intervention Services). By contrast our sample differs since they represent newly recorded cases in primary care throughout the UK. Not all patients with SMI are diagnosed or treated in secondary care this may be a reason why the rate is higher in this study than studies originating from hospital settings. It may also be the case that an individual with SMI is more likely to be registered with a GP than the general population. The other existing study to use primary care data [13] used the General Practice Research Database (GPRD) to identify a cohort with first onset of psychotic illness and found an incidence of 65 per 100,000 PYAR. However they did not apply the rigorous inclusion/exclusion criteria of our study.

**Table 4:** UK based population incidence estimates using data collected between 1995–2010.

First Author	Publication year	Data collection years	Setting	Number of patients	Incidence (per 100,000 PYAR)	95% CI (per 100,000 PYAR)
**ALL SMI**						
**Hardoon**	**2013**	**2000–2010**	**ALL UK**	**8571**	**46.4**	**45.4–47.4**
Reay	2010	1999–2005	Northumberland	441	31.0	27.2–33.2
Coid	2008	1997–1999	East London	484	58.4	53.4–63.9
Gould	2006	2002	North London	111	30.0	24.9–36.1
Kirkbride	2006	1997–1999	London/Bristol/Nottingham	568	34.8	32.1–37.8
Mahmood	2006	2001–2005	South London	303	100.0	N/A
Proctor	2004	1998–2001	Northumberland	227	30.4	26.4–34.3
Singh	2003	2000	West/South London	295	21.0	18.7–23.5
Scully	2002	1995–2000	County Cavan, Ireland	69	18.7	14.6–23.7
Rowlands	2001	1999	Derbyshire	84	36.0	29.1–44.6
**Schizophrenia**						
**Hardoon**	**2013**	**2000–2010**	**ALL UK**	**1694**	**9.2**	**8.7–9.6**
Reay	2010	1999–2005	Northumberland	60	17.0	15.0–19.0
Coid	2008	1997–1999	East London	268	32.4	28.7–36.5
Kirkbride	2006	1997–1999	London/Bristol/Nottingham	209	12.0	11.2–14.7
Proctor	2004	1998–2001	Northumberland	128	17.1	6.4–34.3
Scully	2002	1995–2000	County Cavan, Ireland	35	9.5	6.6–13.2
**Bipolar Disorder**						
**Hardoon**	**2013**	**2000–2010**	**ALL UK**	**2762**	**15.0**	**14.4–15.5**
Reay	2010	1999–2005	Northumberland	44	3.2	2.4–4.4
Lloyd	2005	1997–1999	London/Bristol/Nottingham	75	4.6	2.7–5.8
Scully	2002	1995–2000	County Cavan, Ireland	8	2.2	0.9–4.3

Adapted from [8].

Our recorded rate of schizophrenia (8.2 per 100,000 PYAR) was lower than in population incidence studies (Table 4), but it is possible that many patients fulfilling criteria for schizophrenia were coded in the other non-organic psychosis group; this diagnosis has become increasingly common with development of Early Intervention Services who are wary of diagnosing schizophrenia early in the illness [30]. This trend has also been recognised in GPRD [13]. The data are consistent with established epidemiological trends for schizophrenia; namely that it is more common in men than women [31], most commonly diagnosed in the 16–25 year age band [32], and an increasing incidence with an increase in social deprivation [12]. However after adjustment the difference by urbanicity was non-significant [31].

The recorded rate of bipolar disorder (13.6 per 100,000 PYAR) was higher than in other UK studies, but contemporary UK incidence data on bipolar disorder is limited (Table 4) and recent studies have low patient numbers (less than 100 cases). In the US the incidence of bipolar disorder has been found to be as high as 500 persons per 100 000 PYAR [33]. In studies of European populations the incidence of those who sought treatment for bipolar disorder (15 years of age or older) varied from 9.2 to 15.2 males and from 7.4 to 32.5 females per 100 000 PYAR [34]–[36] which is more consistent with our findings. Concerns about increasing diagnosis of bipolar disorder over time [37], [38] are not borne out by our results. Bipolar disorder coding followed patterns seen in community samples: more common in women [39](perhaps representing more frequent bipolar II disorder and increased treatment), later diagnosis than schizophrenia [40], and similar increases in deprived [41]and urban populations (though this was not significant after adjustment in our sample) [42].

“Other non-organic psychosis” codes are the most common method of recording psychosis in primary care, which may reflect hesitancy to assign a diagnosis that could be considered stigmatising. This may be particularly true for patients who do not initially show a clear presentation of bipolar disorder or schizophrenia and for whom a firm diagnosis may be premature. Whilst many of these patients may ultimately be diagnosed with bipolar disorder or schizophrenia, others may show no further symptoms or receive a different diagnosis such as drug-induced psychosis or schizoaffective disorder. The “other non-organic psychosis” group is unusual in that in males it follows age of onset patterns seen in schizophrenia, but in females appears to be picking up other types of diagnosis (such as delusional disorder or “paraphrenias”) being most common over the age of 75. Of the cohort initially assigned an “other non-organic psychosis” diagnosis 8.0% were eventually coded as having schizophrenia. The high stability (90.3%) of SMI diagnosis is in keeping with previous studies [43], [44].

Early intervention Services in the UK were established with the expectation that they would be providing care to 15 new patients per 100,000 population each year [14].This study suggests the number of patients fulfilling criteria could be as high as 58.1 per 100,000 PYAR, and therefore that service provision to this vulnerable cohort may not be sustainable.

### Strengths and Limitations

A key strength of this study is the large size of the population sample (over 4 million patients) enabling precise estimates of rates of recording in primary care. Furthermore, THIN covers the whole UK and is broadly demographically representative of UK primary care patients [18].

There are multiple challenges in estimating incidence from dynamic, longitudinal GP records. We defined our cases by Read code diagnosis as we were looking specifically at GP-recorded SMI, so these patients may not be regarded as “cases” in terms of standardised diagnostic criteria (ie ICD-10 or DSM-5) however previous research has found the diagnosis of psychosis (based on clinician reported Read codes) to be valid [17]. Because of the nature of the data, we can only state that these are newly *recorded* cases, rather than true incident cases of SMI; however we attempted to reduce the chance of prevalent cases being recorded as new by excluding those registered for less than one year and excluding Read codes suggestive of chronic illness (e.g. “chronic schizophrenia”).

People who received a prescription for an antipsychotic medication without an SMI code being entered were excluded, as it was unclear what the GP's working diagnosis was in such cases. This group would include those prescribed antipsychotic medication for another indication, such as behavioural disturbance, dementia or severe obsessive compulsive disorder.

The data are limited to GP-recorded SMI in-practice attendees, which reflect incidence, presentation and recording at a practice level only. Therefore it is possible that some individuals (especially younger men [45]) have been missed, as they are not registered with a GP which would be likely to disproportionately reduce the total population at risk in comparison to the number of cases (as individuals with SMI are highly likely to be registered with a GP). There is also a possibility that the patients detected (especially in older age groups) had a previous diagnosis of SMI, but that this was not recorded when they later moved into a THIN contributing practice. We attempted to limit this by excluding individuals coded within one year of registration. There may be SMI symptoms coded in free text, which would suggest our findings may be an underestimate of the true burden of disease in primary care. The rates we found are however, higher than that found in other studies identifying people mainly in secondary care settings. A number of patients (4,151) were coded as being included on the general practice SMI register, but were not picked up by our Read code list search, and as a result we excluded them from the analysis. We have not determined why these patients are included on the SMI register without also having an SMI diagnosis, but it may be that GPs have also coded as SMI those patients with mental health problems who require a lot of input but don’t truly meet the defined criteria of schizophrenia, bipolar disorder or other psychotic illness (such as chronic recurrent depression, anxiety or personality disorder). There are no formal checks made on who is added to the SMI register and there is evidence that there was confusion around the definition early on [1]. Peaks in the recording of each specific diagnosis in the year QOF was introduced (2004) suggest that GP's did a ‘catch-up’ of recording and that some of these were actually prevalent cases rather than newly diagnosed.

Another limitation is the lack of recording of ethnicity in primary care. Recording of ethnicity has improved since 2005, in particular for newly registered patients. However, there is still a large proportion without information [46]. Therefore, we did not make an attempt to establish whether certain ethnic groups were more likely to have a record of psychotic illnesses as shown elsewhere [8].

## Conclusions

We have shown 1) that the overall rate of new recording of SMI in THIN is slightly higher than SMI incidence in UK community epidemiological studies, 2) that rates of specific diagnosis differ, but that they are in keeping with international estimates and show changes in “labelling” of specific SMIs, and 3) that, after considering age/sex interaction the socio-demographics of our cohort fit established patterns. In combination these factors confirm the suitability of THIN data as a resource for future research into SMI. A peak in recording occurred in 2004 which may in part reflect updating of primary care records at the time of the introduction of QOF for SMI. After the introduction of QOF, rates remained stable at around 40 new cases per 100,000 person years, more likely representing the true numbers of new cases in primary care. Schizophrenia is more rarely coded than population estimates of incidence in existing studies, and is reducing over time. Bipolar disorder is more commonly coded but rates remained relatively stable over the study period. Our findings suggest that rates of SMI among the 16 to 35 year age group might be higher than that anticipated in development of Early Intervention Services.
